# Functional Cross-Talk between the α_1_- and β_1_-Adrenergic Receptors Modulates the Rapidly Activating Delayed Rectifier Potassium Current in Guinea Pig Ventricular Myocytes

**DOI:** 10.3390/ijms150814220

**Published:** 2014-08-14

**Authors:** Di Xu, Sen Wang, Ting-Ting Wu, Xiao-Yan Wang, Jin Qian, Yan Guo

**Affiliations:** Department of Geriatric Cardiology, the First Affiliated Hospital of Nanjing Medical University, Nanjing 210029, China; E-Mails: ws20041087@hotmail.com (S.W.); cecilywoo@163.com (T.-T.W.); amy_njmu@126.com (X.-Y.W.); qianjinxxg@163.com (J.Q.); guoyan51@hotmail.com (Y.G.)

**Keywords:** adrenergic receptors, potassium current, cross-talk, arrhythmia

## Abstract

The rapidly activating delayed rectifier potassium current (*I*_Kr_) plays a critical role in cardiac repolarization. Although *I*_Kr_ is known to be regulated by both α_1_- and β_1_-adrenergic receptors (ARs), the cross-talk and feedback mechanisms that dictate its response to α_1_- and β_1_-AR activation are not known. In the present study, *I*_Kr_ was recorded using the whole-cell patch-clamp technique. *I*_Kr_ amplitude was measured before and after the sequential application of selective adrenergic agonists targeting α_1_- and β_1_-ARs. Stimulation of either receptor alone (α_1_-ARs using 1 μM phenylephrine (PE) or β_1_-ARs using 10 μM xamoterol (Xamo)) reduced *I*_Kr_ by 0.22 ± 0.03 and 0.28 ± 0.01, respectively. The voltage-dependent activation curve of *I*_Kr_ shifted in the negative direction. The half-maximal activation voltage (*V*_0.5_) was altered by −6.35 ± 1.53 and −1.95 ± 2.22 mV, respectively, with no major change in the slope factor (*k*). When myocytes were pretreated with Xamo, PE-induced reduction in *I*_Kr_ was markedly blunted and the corresponding change in *V*_0.5_ was significantly altered. Similarly, when cells were pretreated with PE, Xamo-induced reduction of *I*_Kr_ was significantly attenuated. The present results demonstrate that functional cross-talk between α_1_- and β_1_-AR signaling regulates *I*_Kr_. Such non-linear regulation may form a protective mechanism under excessive adrenergic stimulation.

## 1. Introduction

The human ether-a-go-go related gene (*hERG*) encodes the pore-forming subunit of the voltage-dependent potassium channel that conducts the rapidly activating delayed rectifier potassium current (*I*_Kr_) [1,2]. *I*_Kr_ exhibits slow activation and deactivation kinetics, coupled with rapid voltage-dependent inactivation and recovery from inactivation. These unique features of *I*_Kr_ make it a critical repolarizing current in ventricular myocytes. Indeed, disruptions in *I*_Kr_ have been shown to underlie abnormal action potential repolarization and to promote arrhythmogenic early afterdepolarization triggers, leading to sudden death in congenital and acquired cardiovascular disorders, including the long QT syndrome [3].

Numerous studies have documented the regulation of ion currents by neurotransmitters and hormones. Acute activation of β_1_-adrenergic receptors (β_1_-ARs), classically coupling with G_s_-proteins, results in adenylate cyclase and thereby increases cyclic AMP (cAMP). This promotes protein kinase A (PKA)-mediated phosphorylation of four recognized serine residues (S283, S890, S895, and S1137) on the *hERG* channel. This, in turn, reduces *I*_Kr_ density [4,5,6,7]. On the other hand, acute stimulation of α_1_-adrenergic receptors (α_1_-ARs) leads to activation of phosphatidyl inositol-specific phospholipase C (PLC). The PLC substrate phosphatidyl-4,5-bisphosphate (PIP_2_), a membrane phospholipid, is hydrolyzed to generate the intracellular second messengers, 1,4,5-inositol trisphosphate (IP_3_) and diacylglycerol (DAG). IP3 induces mobilization of intracellular calcium, while DAG acts as a physiological activator of protein kinase C (PKC), a serine-threonine-dependent kinase known to phosphorylate the *hERG* channel and reduce *I*_Kr_ density [7,8,9,10].

The complexity of the adrenergic regulation of *hERG* is underscored by the fact that during emotional or physical stress, catecholamines bind to multiple adrenoreceptors rather than act selectively on specific ones. Rorabaugh *et al.* demonstrated that stimulation of α_1_-ARs could down-regulate β_1_-AR-mediated inotropy in the mouse heart [11]. Moreover, cross-activation of PKA, PIP2 and PKC can regulate *I*_Na_ [12], the slowly activating delayed rectifer K^+^ current (*I*_Ks_) [13,14] and *I*_Kr_ [4,9,15,16]. Indeed, adrenergic signal transduction pathways may exert complex inhibitory effects on cardiac *hERG*/*I*_kr_ currents via multiple mechanisms that potentially involve the intracellular second messenger cAMP, protein kinases A and C, and possibly other regulatory components of the *hERG* macromolecular complex, including minK, MiRP1, and 14-3-3. Signaling “cross talk” between α_1_- and β_1_-adrenergic cascades might also be involved, although direct experimental evidence is lacking.

We hypothesized that due to complex cross-talk between α_1_- and β_1_-adrenergic cascades, *I*_Kr_ would exhibit non-linear responses to combined adrenergic stimulation. In other words, the combined activation of both pathways in terms of *I*_Kr_ inhibition would not reflect the sum of the individual effects of stimulating either axis alone. To address this hypothesis, we designed two experimental groups, which are referred to as P_pre_ + X and X_pre_ + P. In the P_pre_ + X group, 1 μM Phenylephrine (PE) was applied for 9 min resulting in a steady state *I*_Kr_ inhibition, followed by co-application of 10 μM xamoterol (Xamo) and 10 μM PE for another 9 min. Conversely, in the X_pre_ + P group, 10 μM Xamo was pre-applied for 9 min, followed by simultaneous application of both 1 μM PE and 10 μM Xamo for another 9 min. Hence, we were able to compare the reductions and shifts in activation curves of *I*_Kr_ caused by exposure to PE alone, Xamo alone, and PE plus Xamo. More importantly, we compared PE-induced *I*_Kr_ reduction in the presence of Xamo, and Xamo-induced *I*_Kr_ reduction in the presence of PE, as well as shifts in the activation curve of *I*_Kr_ under varying conditions.

## 2. Results and Discussion

### 2.1. Confirmation of the Rapidly Activating Delayed Rectifier Potassium Current (I_Kr_) Identity and Stability

**Figure 1:**
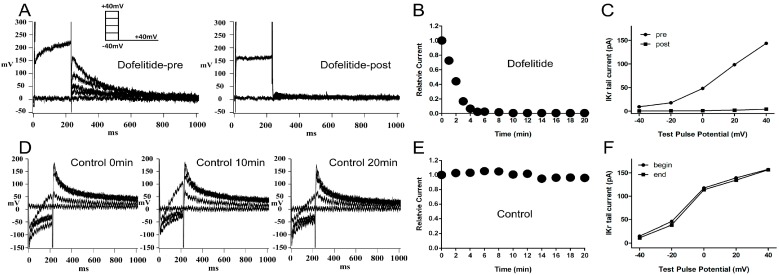
Confirmation and stability of *I*_Kr_ current. (**A**) Representative original *I*_Kr_ tail currents recorded from the same left ventricular guinea pig myocyte before and 20 min after application of dofetilide (1 μM); (**B**) shows the representative trace of time-dependence of the relative current reduction by dofetilide; (**C**) The curve of *I*_Kr_ tail currents at different test pulse potential (I–V curve) before and after dofetilide application. (**A**–**C**) demonstrate the lack of contamination of the tail current by non-*I*_Kr_ currents under our experimental conditions; (**D**) Typical original *I*_Kr_ tail currents recorded over a 20-min period from a myocyte; (**E**,**F**) show the representative traces of time-dependence of the relative current and the I–V curve during myocyte superperfusion with control extracellular solution. (**D**–**F**) demonstrate that the *I*_Kr_ tail currents were stable within a timeframe of 20 min. Protocol I: holding potential −40 mV, test pulses from −40 to +40 mV in 20 mV increments (duration 225 ms), return pulse to −40 mV (duration 775 ms) to measure *I*_Kr_ tail currents. Unless specified otherwise, all *I*_Kr_ tail currents were induced by protocol I.

When 1 μM dofetilide, a specific *I*_Kr_ blocker, was applied to the extracellular solution, the *I*_Kr_ tail current was almost completely eliminated (Figure 1A). The *I*_Kr_ tail current at the return pulse of −40 mV after depolarizing to +40 mV, exhibited a marked time-dependent decrease to negligible levels within 10 min (Figure 1B). We plotted the tail current-voltage relationship at 0 and 20 min during application of dofelitide (Figure 1C) and found that the *I*_Kr_ tail current was completely abolished by dofetilide. These data indicated that no other contaminating currents contributed to the *I*_Kr_ tail current under our experimental conditions.

The *I*_Kr_ tail current at the return pulse of −40 mV from a test potential of +40 mV, was stable for at least 20 min of perfusion with control extracellular solution (Figure 1D,E). The tail current-voltage curves at 0 and 20 min were almost identical (Figure 1F) indicating presence of minimal tail current rundown under our experimental conditions.

### 2.2. Cell Capacitance and Basic Gating Data in Different Groups

**Figure 2:**
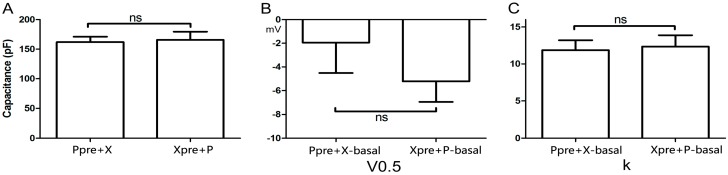
Cell capacitance and basic gating data in different groups. (**A**) Cell capacitance in the P_pre_ + X and X_pre_ + P groups, showing no significant difference (*n* = 7); (**B**,**C**) The half-maximal activation voltage (*V*_0.5_) and the slope factor (*k*) of myocytes at basal conditions in the two groups also exhibit no significant difference (*n* = 7). These data indicate that myocytes used in either group exhibit comparable properties at baseline. “ns” indicates “not significant”.

The two groups (P_pre_ + X and X_pre_ + P) that were defined above were studied. Myocyte size, measured as cell capacitance, was comparable (*p* = 0.82, Figure 2A) between groups (161.80 ± 9.23 pF for P_pre_ + X and 165.70 ± 13.93 pF for X_pre_ + P, *n* = 7 each). Two key characteristics of the activating curve (*V*_0.5_ and *k*) were measured in both groups during exposure of myocytes to the control bath solution. As shown in Figure 2B, *V*_0.5_ was not significantly different between the P_pre_ + X (−1.93 ± 2.58 mV) and the X_pre_ + PE (−5.22 ± 1.73 mV) groups (*p* = 0.31, *n* = 7). Similarly, *K* was also comparable (*p* = 0.82, Figure 2C) between groups (11.92 ± 1.33 for P_pre_ + X and 12.35 ± 1.54 for X_pre_ + P).

### 2.3. Cellular Electrophysiology Data of the P_pre_ + X Group

**Figure 3:**
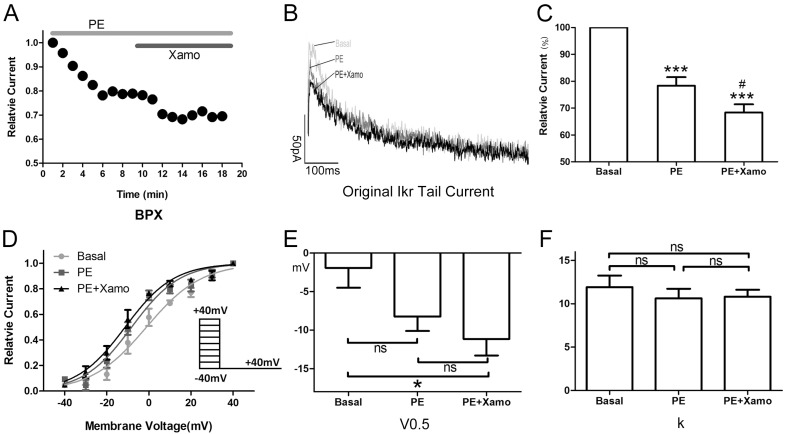
Cellular electrophysiology data of the P_pre_ + X group; (**A**) shows the representative traces of time-dependence of the relative current reduction by phenylephrine (PE, 1 μM) and combined PE plus Xamo (Xamo, 10 μM); (**B**) Typical original *I*_Kr_ tail currents recorded at return pulse after depolarizing to +40 mV at basal conditions and after application of PE and PE + Xamo; (**C**) Comparison of the relative current at baseline, following 9-min exposure to PE, and following 9-min exposure to PE + Xamo (*n* = 7; *** *p* < 0.001, *vs.* basal; # *p* < 0.05, *vs.* PE); (**D**) The plots of *I*_tail_/*I*_tail.max_ *vs.* membrane voltage at three different conditions, fit with the single-power Boltzmann equation: *I*_tail_ = *I*_tail.max_/[1 + exp(*V*_0.5_ − *V*)/*k*], reflecting the activation kinetics. Here, *I*_Kr_ tail currents were induced by protocol II: holding potential −40 mV, test pulses from −40 to +40 mV in 10 mV increments (duration 225 ms), return pulse to −40 mV (duration 775 ms); (**E**,**F**) *V*_0.5_ and *k* of the myocytes measured at three different conditions (*n* = 7). “ns” indicates “not significant”; * indicates *p* < 0.05).

When myocytes were exposed to 1 μM PE alone, the *I*_Kr_ tail current decreased rapidly, reaching a steady-state nadir within 4–6 min (Figure 3A,B). After 9 min of PE exposure, *I*_Kr_ tail current density measured at +40 mV decreased from 0.80 ± 0.04 to 0.62 ± 0.01 pA/pF, which means *I*_Kr_ tail current decreased to (78.33 ± 3.19)% compaired to the basal *I*_Kr_ tail current (*n* = 7; *p* < 0.001; Figure 3C). Voltage-dependent activation of *I*_Kr_ exhibited a trend towards a shift in the negative direction (Figure 3D), with the half-maximal activation voltage (*V*_0.5_) changing from −1.93 ± 2.58 to −8.24 ± 1.88 mV (*n* = 7; *p* = 0.06; Figure 3E). Similarly, the change in slope factor (*k*) from 11.92 ± 1.33 to 10.63 ± 1.10 (*n* = 7; *p* = 0.42; Figure 3F) did not reach statistical significance. Co-application of Xamo decreased *I*_Kr_ tail current density even further to 0.54 ± 0.01 pA/pF. This additional reduction in current density was significant when compared to baseline control levels (*n* = 7; *p* < 0.001; Figure 3C) as well as to those achieved with PE alone (*n* = 7; *p* < 0.05; Figure 3C). As such, during α_1_-AR activation, the Xamo-mediated decrease was only 0.13 ± 0.01 (*n* = 7; Figure 7). The voltage-dependent activation curve of *I*_Kr_ remained unchanged compared with PE alone (*n* = 7, Figure 3D), with *V*_0.5_ changing to −11.15 ± 2.15 mV, (*n* = 7; *p* < 0.05, compared to the basal *V*_0.5_; Figure 3E), and *k* changing to 10.82 ± 0.80 (*n* = 7; *p* = 0.49, compared to the basal *k*; Figure 3F).

**Figure 4:**
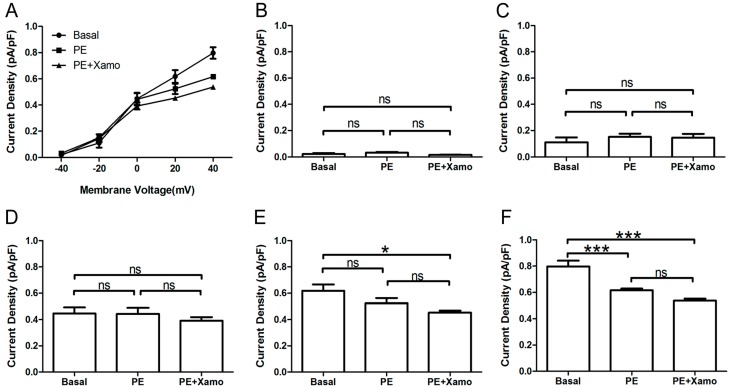
*I*_Kr_ tail currents at different depolarization levels in group P_pre_ + X group. (**A**) *I*_Kr_ tail current densities (pA/pF) measured at different membrane voltages before and after treatment with PE alone or PE + Xamo; (**B**–**F**) Comparison of *I*_Kr_ tail current densities at baseline and following PE alone and PE + Xamo treatment. *I*_Kr_ tail currents were seperately measured when the test pulse was at −40, −20, 0, +20, and +40 mV (*n* = 7). “ns” indicates “not significant”; * *p* < 0.05; *** *p* < 0.001.

Shown in Figure 4 are *I*_Kr_ tail current density measurements obtained over a wide range of test voltages before and after treatment of myocytes with PE alone or combined PE + Xamo. Neither PE alone nor PE + Xamo were sufficient to alter *I*_Kr_ tail current density compared to pre-treatment levels at −40, −20, and 0 mV. Interestingly, at +20 mV, the combined treatment (PE + Xamo) but not PE alone was associated with a significant reduction in tail current density (*n* = 7; *p* < 0.05; Figure 4E). Finally, at +40 mV, both PE alone and PE + Xamo were sufficient to elicit significant reductions in tail current density as compared to basal pre-treatment levels (*n* = 7; each *p* value was less than 0.001; Figure 4F).

### 2.4. Cellular Electrophysiological Data of the X_pre_ + P Group

**Figure 5:**
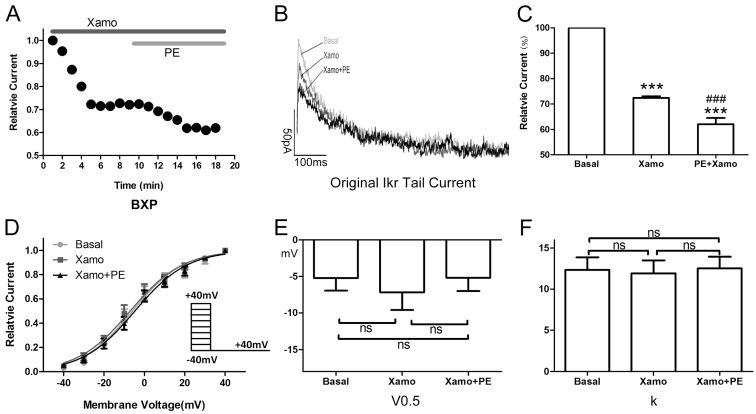
Cellular electrophysiology data of the X_pre_ + P group. (**A**) shows the representative traces of time-dependence of the relative current reduction by xamoterol (Xamo, 10 μM) and combined Xamo plus phenylephrine (PE, 1 μM); (**B**) Typical original *I*_Kr_ tail currents recorded at return pulse after depolarizing to +40 mV at basal conditions and after application of Xamo and Xamo + PE; (**C**) Comparison of the relative current at baseline, following 9-min exposure to Xamo, and 9-min exposure to Xamo + PE (*n* = 7; *** *p* < 0.001, *vs.* basal; ### *p* < 0.001, *vs.* Xamo); (**D**,**E**,**F**) The plots of *I*_tail_/*I*_tail.max_ *vs.* membrane voltage under three different conditions, fitting with Blotzmann equation, and *V*_0.5_ and *k* of the myocytes measured under three different conditions (*n* = 7). “ns” indicates “not significant”.

When exposed to 10 μM Xamo alone, the *I*_Kr_ tail current decreased rapidly, reaching steady-state after 4–6 min (Figure 5A,B). At the end of 9 min of drug exposure, *I*_Kr_ tail current density was reduced from 1.09 ± 0.07 to 0.80 ± 0.05 pA/pF at +40 mV, which means *I*_Kr_ tail current decreased to (72.43 ± 0.64)% compaired to the basal *I*_Kr_ tail current (*n* = 7; *p* < 0.001; Figure 5C). In other words, the Xamo-induced decrease was about 0.28 ± 0.01 (*n* = 7; Figure 7). The voltage-dependent curve of *I*_Kr_ activation exhibited a modest shift in the negative direction (Figure 5D), with *V*_0.5_ changing from −5.22 ± 1.73 to −7.17 ± 2.43 mV (*n* = 7; *p* = 0.50; Figure 5E), while *k* shifted from 12.35 ± 1.54 to 11.93 ± 1.57 (*n* = 7; *p* = 0.85; Figure 5F). Co-application of 1 μM PE decreased the *I*_Kr_ tail current further within 5 min (Figure 5A,B). The *I*_Kr_ tail current density changed to 0.69 ± 0.06 pA/pF at +40 mV, and was about (62.03 ± 2.43)% of the basal *I*_Kr_ tail current (*n* = 7; *p* < 0.001; Figure 5C), and (85.56 ± 2.94)% of the *I*_Kr_ tail current measured with 10 μM Xamo alone (*n* = 7; *p* < 0.001; Figure 5C). As such, under conditions of β_1_-adrenergic activation, the PE-induced decrease in tail current amplitude was only 0.14 ± 0.03 (*n* = 7; Figure 7) of the basal level. The voltage-dependent curve of *I*_Kr_ activation almost remained unchanged (*n* = 7, Figure 5D), with *V*_0.5_ changing to −5.19 ± 1.83 mV (*n* = 7; *p* = 0.99 *vs.* basal group and *p* = 0.50 *vs.* Xamo group; Figure 5E), and *k* changing to 12.52 ± 1.44 (*n* = 7; *p* = 0.94 *vs.* basal group and *p* = 0.79 *vs.* Xamo group; Figure 5F).

**Figure 6:**
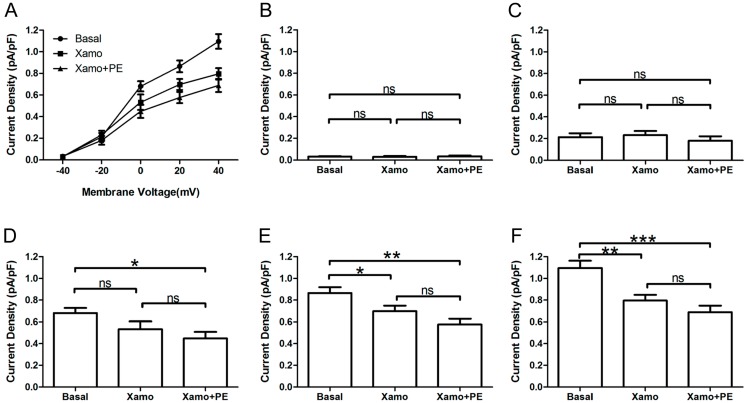
*I*_Kr_ tail currents at different depolarization level in group X_pre_ + P group. (**A**) The *I*_Kr_ tail current densities (pA/pF) at different membrane voltages under three different conditions; (**B**–**F**) Comparison of *I*_Kr_ tail current densities under three different conditions, at return pulse after depolarizing to −40, −20, 0, +20, and +40 mV separately (*n* = 7). “ns” indicates “not significant”; * *p* < 0.05; ** *p* < 0.01; *** *p* < 0.001).

Furthermore, the *I*_Kr_ tail currents at different depolarization levels were tested and compared before and after exposure of myocytes to PE alone or PE + Xamo (Figure 6A). As shown in Figure 6B,C, the *I*_Kr_ tail currents measured at −40 and −20 mV did not exhibit statistical differences across groups (*n* = 7). At 0 mV, exposure of myocytes to combined PE + Xamo but not PE alone resulted in a significant reduction in current density compared to basal pretreatment levels (*n* = 7; *p* < 0.05; Figure 6D). In contrast, at +20 and the *I*_Kr_ tail current was statistically different to basal one at +20 and +40 mV, both PE alone as well as PE + Xamo were sufficient to elicit significant decreases in current density as compared to basal pretreatment levels (*n* = 7; all *p* value was less than 0.05; Figure 6E,F).

### 2.5. Comparison of Effects by Different Adrenergic Activation

**Figure 7:**
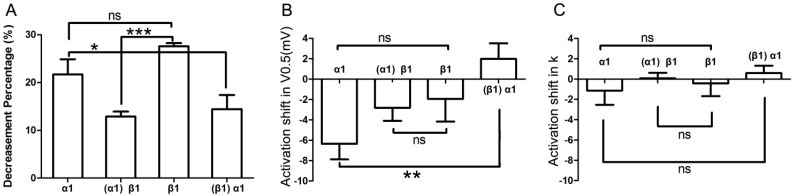
Comparison of effects by different adrenergic activation. (**A**) Comparison of the decrease in *I*_Kr_ tail current after application of various AR agonists. Column α_1_ represents the percent decrease in *I*_Kr_ tail current by application of α_1_-AR agonist alone; and column (α_1_)β_1_ represents the β_1_-AR mediated percent decrease of *I*_Kr_ after pre-activation of α_1_-AR; similarly, column β_1_ stands for β_1_-AR alone; column (β_1_)α_1_ stands for α_1_-AR mediated percent inhibition of *I*_Kr_ after pre-stimulation of β_1_-AR (*n* = 7); (**B**,**C**) Comparison of activation shifts in corresponding *V*_0.5_ and *k* under different conditions. The four columns, α_1_, (α_1_)β_1_, β_1_, and (β_1_)α_1_ represent conditions of α_1_-AR activation alone, β_1_-AR activation in presence of α_1_-AR activation, β_1_-AR activation alone, and α_1_-AR activation in presence of β_1_-AR, respectively (*n* = 7). “ns” indicates “not significant”; * *p* < 0.05; ** *p* < 0.01; *** *p* < 0.001.

As shown in Figure 7A, α_1_-adrenergic activation-induced inhibition of *I*_Kr_ tail current was less than β_1_-adrenergic activation-induced inhibition (0.22 ± 0.03 & 0.28 ± 0.01), but the difference between the two groups did not reach statistical significance (*n* = 7; *p* = 0.08). Similarly, the minor activation shifts in terms of *V*_0.5_ and *k* values were comparable across groups (*n* = 7; Figure 7B,C). However, the α_1_-AR induced inhibition of *I*_Kr_ tail current significantly differed in cells that were pre-incubated with the β_1_-AR agonist compared to control (*i.e.*, otherwise unstimulated myocytes (*n* = 7; 0.22 ± 0.03 & 0.14 ± 0.03, *p* < 0.05; Figure 7A). Moreover, while the activation shift in terms of *V*_0.5_ induced by concomitant α_1_- and β_1_-AR stimulation was significantly different (*n* = 7; (−6.35 ± 1.53) & (1.98 ± 1.52) mV, *p* < 0.01; Figure 7B) compared to α_1_-AR stimulation alone, the corresponding *k* values did not show statistical differences between the two groups (*n* = 7; −1.13 ± 1.42 & 0.60 ± 0.73; Figure 7C). Similarly, the β_1_-AR activation-induced inhibition of *I*_Kr_ tail current significantly differed between unstimulated (control) *vs.* α_1_-AR stimulated myocytes (*n* = 7; 0.28 ± 0.01 & 0.13 ± 0.01, *p* < 0.001; Figure 7A). Finally, the activation shift in terms of *V*_0.5_ and *k* values did not show any statistically significant differences (*n* = 7; (−1.95 ± 2.22) & (−2.82 ± 1.28) mV, and (−0.42 ± 1.26) & (−0.07 ± 0.54) mV; Figure 7B,C).

### 2.6. Discussion

The main findings of the present report are as follows: (1) acute activation of α_1_-ARs produce comparable effects on *I*_Kr_ tail current density to β_1_-ARs; (2) acute activation of α_1_-AR in the presence of β_1_-AR activation elicits a minor decrease in *I*_Kr_ tail current, which is statistically different from that achieved by α_1_-AR activation alone; (3) similarly, acute activation of β_1_-AR in the presence of α_1_-AR activation induces a very small decrease in *I*_Kr_ tail current, which again is statistically different from that achieved by β_1_-AR activation alone. The blunted *I*_Kr_ response to concomitant adrenergic activation is suggestive of a protective feedback regulatory mechanism that acts to maintain the *I*_Kr_ tail current density in the wake of excessive catecholamine stress, modeled *in vitro* in our study as combined PE and Xamo exposure.

In the present study, the finding that PE or Xamo is able to decrease the *I*_Kr_ tail current via stimulation of α_1_-ARs or β_1_-ARs is consistent with previous reports [5,7,9,17,18,19,20], including our own published work [21] in HEK-293 cells, CHO cells, *Xenopus* oocytes, and native ventricular cardiomyocytes. The *I*_Kr_ tail current exhibits a concentration-dependent decrease after acute stimulation of α_1_-ARs or β_1_-ARs within 4–6 min, reaching steady-state levels within 7–9 min. Our choice of drug concentrations was guided by previous work, in which we determined the IC_50_ values of PE and Xamo to be approximately 0.9 and 6.4 μM, respectively [22]. The *I*_Kr_ tail current induced by depolarization to +40 mV was 0.78 ± 0.03 or 0.72 ± 0.01 of the basal *I*_Kr_ tail current after application of 1 μM PE or 10 μM Xamo, respectively (*p* < 0.05). The *I*_Kr_ tail current activation curve exhibited a minor shift in the repolarizing direction in response to α_1_-AR stimulation that was comparable to that achieved by β_1_-AR activation (Figure 5B,C), suggesting that the gating kinetics of the *hERG* channel was not markedly affected by acute α_1_-AR or β_1_-AR stimulation separately. Characterized by slow activation and deactivation kinetics, and rapid voltage-dependent inactivation and recovery from inactivation kinetics, *I*_Kr_ encoded by *hERG* is indeed a critical component of action potential repolarization in both atrial and ventricular myocytes of most species, including humans [2,3,23,24]. Thus, excessive *I*_Kr_ inhibition causes marked repolarization delays that result in prolongation of the action potential at the cellular level, and the QT-interval of the electrocardiogram at the body surface level; thereby promoting the incidence of arrhythmogenic early afterdepolarizations, and polymorphic ventricular tachycardia. As such, our *in vitro* findings have direct relevance to clinical scenarios, in which patients with inherited or acquired long QT syndrome (LQTS) experience stress-related arrhythmias, in many cases leading to sudden cardiac death.

Of note, co-application of both β_1_- and α_1_-AR agonists resulted in a relatively small additional inhibitory effect on *I*_Kr_ tail current compared to the selective activation of either receptor alone. Importantly, however, the decrease in *I*_Kr_ tail current produced by PE in the presence of Xamo was significantly different from that achieved by α_1_-AR activation alone (0.14 ± 0.03 *vs.* 0.22 ± 0.03, *p* < 0.05). Similarly, the decrease in *I*_Kr_ tail current by β_1_-AR stimulation in the wake of α_1_-AR activation was significantly different from that produced by β_1_-AR activation alone (0.13 ± 0.01 *vs.* 0.28 ± 0.01, *p* < 0.001). This suggests that pre-activation of β_1_-ARs markedly suppresses the inhibitory effect of α_1_-AR activation on *I*_Kr_ tail current, and that pre-activation of α_1_-ARs produces an even stronger modulatory effect on the inhibition of *I*_Kr_ by β_1_-AR. Indeed, pre-activation of one adrenoreceptor subclass dramatically restricts the inhibitory effect of the other subclass on *I*_Kr_ tail current. As such, there appears to be significant cross-talk in the regulation of *I*_Kr_ by acute adrenergic stimulation of AR receptors. We propose that this tight regulatory mechanism acts to protect against excessive *I*_Kr_ inhibition, and therefore action potential prolongation, under conditions of extreme stress and associated catecholamine release.

Acute nonselective α_1_-AR activation suppresses the positive inotropic effect of β-AR activation and associated cAMP generation [25]. Under certain conditions, α_1_- and β-AR signaling pathways exhibit synergistic effects [26]. Moreover, α_1_- and β-AR interactions modulate the L-type calcium current [27], and sustained activation of PKC-epsilon leads to a blunted response of the current to α_1_- and β-AR signaling [28]. In addition, PKC activation cross-activates PKA to modulate the cardiac Na^+^ current [12]. Moreover, both PKA and PKC regulate the cardiac *I*_Ks_ in a mutually exclusive manner [13], and the channel phosphorylation by PKA cross-activates PLC-dependent regulation, which activates downstream PKC [14]. Therefore, the linear signaling paradigm has given way to a complex multidimensional “signalome” in which an individual adrenoceptor can dynamically couple to multiple signaling proteins in a temporally and spatially regulated manner resulting in pharmacologically and functionally distinct receptor populations [29,30,31,32]. Remarkably, mechanistic studies focusing on the regulation of ionic currents by AR signaling have been rapidly translated to the bedside [33]. We have previously demonstrated that *I*_Kr_ tail current is inhibited by acute stimulation of α_1_-ARs using PE [21]. Furthermore, PE-mediated inhibition of the current was significantly attenuated by the PKC inhibitor chelerythrine and the PKA inhibitor KT5720, suggesting that activation of PKA and/or PKC may play an important role in mediating the effects of α_1_-ARs on native *I*_Kr_ current in guinea-pig ventricular myocytes. Coincidentally, Thomas and colleagues [9] reported similar phenomena in *Xenopus laevis* oocytes heterologously coexpressing *hERG* channels and human α_1A_-ARs. In contrast, Bian *et al*. [17] found that pretreatment with chelerythrine augmented the inhibitory effects of α_1_-ARs on *I*_Kr_ current. In β_1_-AR regulation of *I*_Kr_, Karle *et al.* found that inhibiting PKA may attenuate the reduction effect of xamotorol on *I*_Kr_ [5], which is consistant with our previous study. Moreover, we also found that PKC inhibition effectively attenuated the effect of Xamo on *I*_Kr_ [34]. This suggests that, in addition to PKA, PKC is also activated as part of the β_1_-AR signaling pathway in the regulation of *I*_Kr_ tail current. Despite that, both PKA and PKC are among the most important signal transduction pathways that modulate the *I*_Kr_/*hERG* current, and they may act in a cross-activation manner, an important issue which will require further investigation.

## 3. Experimental Section

### 3.1. Guinea Pig Ventricular Myocyte Isolation and Electrophysiological Recordings

All experiments were performed in accordance with Animal Care Protocols approved by the Nanjing Medical University Institutional Animal Care and Use Committee (SCXK2002-0031, Experimental animal production license of Jiangsu Province). Single left ventricular myocytes were isolated from the hearts of healthy male adult guinea pigs (300 ± 50 g) as described previously [21]. Cells were transferred to a temperature controlled recording chamber in which they were continuously perfused with the bath solution. Temperature was maintained at 37 ± 0.5 °C by a temperature control system (TC-324B, Warner, Hamden, CT, USA). Whole-cell patch-clamp recordings were performed using an EPC-9 amplifier (HEKA Electronics, Lambrecht/Pfalz, Germany). Pipettes, filled with the pipette solution, had resistances of 1–3 MΩ. The flow rate of the bath solution through the chamber was maintained at 1–2 mL/min.

### 3.2. Cellular Electrophysiology Protocols

*I*_Kr_/*hERG* currents were measured using a two-step protocol, referred to as Protocol I. From a holding potential of −40 mV, currents were activated by a variable test pulse from −40 to +40 mV (in 20 mV steps, 225 ms duration), followed by a return pulse to −40 mV (duration 775 ms) to evoke outward tail currents. Signals were analog-filtered at 2380 Hz, and the sampled interval was 10,000 Hz. The effects of α_1_- and β_1_-AR agonists were investigated on peak tail currents after the return pulse to +40 mV.

An additional two-step protocol (Protocol II) was also used. Specifically, from a holding potential of −40 mV, currents were activated by a variable test pulse from −40 to +40 mV (in 10 mV steps, 225 ms duration), followed by a return pulse to −40 mV (duration 775 ms) to evoke outward tail currents. This protocol was applied to measure each peak tail current at repolarization after different test voltages. Activation curves were fit to a single-power Boltzmann equation: *I*_tail_ = *I*_tail.max_/[1 + exp(*V*_0.5_ − *V*)/*k*], where *I*_tail_ indicates the tail current, *V* represents the test pulse potential, *V*_0.5_ refers to the half-maximal activation voltage, and *k* is the slope factor.

### 3.3. Solutions and Reagents

For *I*_Kr_ recordings, the pipette solution contained (in mmol/L) KCl 140, CaCl_2_ 1, MgCl_2_ 2, HEPES 10, EGTA 11, Na_2_-ATP 5, creatine phosphate (disodium salt) 5, pH 7.2 adjusted with 8 M KOH. The bath solution contained (in mmol/L) NaCl 140, KCl 3.5, CaCl_2_ 1.5, MgSO_4_ 1.4, HEPES 10, pH adjusted 7.4 with 10 M NaOH. Nifedipine (10 μM) and Chromanol 293B (10 μM) were added to the bath solution to block the L-type calcium and delayed rectifier potassium (*I*_Ks_) currents, respectively. Na_2_-ATP, EGTA, creatine phosphate, l-glutamic acid, HEPES, taurine, bovine serum albumin (BSA), nifedipine, chromanol 293B, PE, and dofelitide were purchased from Sigma (St. Louis, MO, USA), collagenase II from Worthington (Lakewood, NJ, USA), and Xamol-hemifumarate from Santa Cruz (Dallas, TX, USA). All other reagents were obtained from Amresco (Solon, OH, USA).

For stock solutions, nifedipine and chromanol 293B were dissolved in dimethyl sulfoxide (DMSO) (10 mM concentration); Xamo in distilled water (10 mM concentration); PE and dofetilide in distilled water (1 mM). All stock solutions were stored at −20 °C except nifedipine which was stored at 4 °C. Before experiments, aliquots of the stock solutions were diluted with the extracellular solution to the desired test concentrations of 1 μM (PE and dofetilide) and 10 μM (Xamo, nifedipine and chromanol 293B). The final concentration of DMSO was less than 0.5% in extracellular solution, which was determined to have no confounding effects on the currents of interest.

### 3.4. Statistical Analysis

Currents were acquired with Pulse + Pulsefit V8.53 (HEKA Electronics, Lambrecht/Pfalz, Germany) and analyzed by SPSS 18.0 software (SPSS Inc., Chicago, IL, USA). Data were expressed as mean ± S.E.M. Statistical significance was evaluated using the unpaired Student’s *t* test. Multiple comparisons were analyzed using one-way analysis of variance (ANOVA), with a *post-hoc* comparison using a Newman–Keuls test. A *p* value less than 0.05 was considered statistically significant.

## 4. Conclusions

*hERG* channel activation is strongly modulated by acute stimulation of α_1_-ARs and β_1_-ARs. Remarkably, stimulation of either receptor class markedly attenuates the inhibitory effects of the other class on *I*_Kr_ Further investigations are required to elucidate the molecular mechanisms that underlie the functional cross-talk or to identify putative intermediate proteins in the signal transduction pathways involved in modulation of *I*_Kr_ current via adrenergic activation.
